# Engineering a New IFN-ApoA-I Fusion Protein with Low Toxicity and Prolonged Action

**DOI:** 10.3390/molecules28248014

**Published:** 2023-12-08

**Authors:** Svetlana Miroshnichenko, Mariya Pykhtina, Anastasiia Kotliarova, Alexander Chepurnov, Anatoly Beklemishev

**Affiliations:** 1 Federal Research Center of Fundamental and Translational Medicine (FRC FTM), Timakova str., 2, 630117 Novosibirsk, Russia; svmiro@yandex.ru (S.M.); alexa.che.purnov@gmail.com (A.C.); ab.beklem-46@yandex.ru (A.B.); 2 N.N. Vorozhtsov Novosibirsk Institute of Organic Chemistry SB RAS, Lavrentiev Ave., 9, 630090 Novosibirsk, Russia; kotliarova@nioch.nsc.ru

**Keywords:** *Pichia pastoris*, yeast-derived IFNα2b (ryIFN), apolipoprotein A-I (ApoA-I), fusion protein, antiviral activity, SARS-CoV-2, cytotoxicity, Tween 20, apoptosis, prolonged half-life

## Abstract

Recombinant human interferon alpha-2b (rIFN) is widely used in antiviral and anticancer immunotherapy. However, the high efficiency of interferon therapy is accompanied by a number of side effects; this problem requires the design of a new class of interferon molecules with reduced cytotoxicity. In this work, IFN was modified via genetic engineering methods by merging it with the blood plasma protein apolipoprotein A-I in order to reduce acute toxicity and improve the pharmacokinetics of IFN. The chimeric protein was obtained via biosynthesis in the yeast *P. pastoris*. The yield of ryIFN-ApoA-I protein when cultivated on a shaker in flasks was 30 mg/L; protein purification was carried out using reverse-phase chromatography to a purity of 95–97%. The chimeric protein demonstrated complete preservation of the biological activity of IFN in the model of vesicular stomatitis virus and SARS-CoV-2. In addition, the chimeric form had reduced cytotoxicity towards Vero cells and increased cell viability under viral load conditions compared with commercial IFN-a2b preparations. Analysis of the pharmacokinetic profile of ryIFN-ApoA-I after a single subcutaneous injection in mice showed a 1.8-fold increased half-life of the chimeric protein compared with ryIFN.

## 1. Introduction

Interferon alpha-2b (IFN) is the most widespread member of the interferon protein family and has many biological effects, such as antiviral, antitumor, immunomodulatory, and antifibrogenic properties. IFN, both alone and in combination treatments with other drugs, is used in the treatment of many diseases, such as viral hepatitis B and C [1] and some types of cancer, including melanoma [2], Kaposi’s sarcoma [3], chronic myeloid leukemia [4], and non-Hodgkin’s lymphoma [5]. The main disadvantages of using recombinant IFN preparations in clinical practice is their short half-live in the patient’s blood (4–8 h) [6] and cytotoxicity [7]. In this regard, to maintain an effective concentration in the blood, frequent injections of rIFN are required, a situation that is often accompanied by various side effects [8], especially from the hematopoietic [9] and nervous systems [10]. The most severe side effects of interferon therapy are cognitive impairment [11] and other neurodegenerative disorders [12,13]. Due to high neurotoxicity, interest in interferon therapy has somewhat decreased despite its highly effective antiviral activity. However, the COVID-19 pandemic has renewed interest in this class of therapy [14]. Thus, the development and implementation of strategies that extend the half-life of IFN and consequently reduce the total amount of injected cytokine and its toxic effect on the body is a significant problem in medical biotechnology. 

Currently, there are several strategies for extending the half-life of therapeutic proteins, including IFN. One of the strategies is pegylation of the protein molecule. Today, there are two commercial preparations of IFN modified by PEG attachment: a linear 12 kDa PEG-conjugated IFN (PEG-Intron) [15] and branched 40 kDa PEG-conjugated IFN (Pegasys) [16]. However, pegylation often results in a heterogeneous and difficult to purify product mixture, low protein yields, and decreased biological activity [17]. In addition, pegylated biomolecules can induce immune responses [18]. Another approach is based on the creation of recombinant fusion proteins containing IFN “fused” with a long-term circulating blood plasma protein. Albumin is the most abundant blood plasma protein used to create chimeric proteins of prolonged action and can significantly extend the half-life of the molecules fused with it. A large number of proteins have been successfully genetically engineered with albumin, and the resulting proteins have demonstrated improved pharmacokinetics [19,20,21,22,23]. However, the creation of chimeric forms of IFN is often accompanied by a decrease in the biological activity of this cytokine [24,25], which may be due to both a violation of the conformation of IFN and a direct effect of HSA (human serum albumin) on the ability of IFN to bind to its receptors. In particular, the IFN-HSA chimeric protein (Albuferon, HGSI, Novartis) allows reducing the frequency of IFN therapy in the treatment of hepatitis C and supports dosing once every 2 or 4 weeks [26] thanks to improved pharmacokinetics, which is a significant improvement in therapy in comparison with therapy using free IFN or pegylated IFN [27]. However, the bioactivity of Albuferon is 10 times lower than wild-type IFN [26]. Considering the benefits of interferon therapy, it is necessary to preserve the functional activity of the cytokine and introduce qualities that reduce its toxicity. Apolipoprotein A-I may be such a candidate for the role of a genetically engineered construct.

In the last decade, blood plasma lipoproteins and their protein components, apolipoproteins, have been widely studied as delivery vehicles for various compounds [28,29,30,31]. Apolipoprotein A-I (ApoA-I) is the main protein component of high-density lipoproteins (HDLs). ApoA-I possesses a number of valuable carrier and protector properties, namely it is amphiphilic and non-immunogenic, circulates in the body for a long time, readily biodegrades, and binds specifically to most cell types due to the presence of SR-BI (scavenger receptor class B type I) receptors [32]. The action of ApoA-I in the body is not limited to the transport role of cholesterol [33]: it also performs many important functions, including antioxidant [34], anti-inflammatory [35], antiviral [36], antiapoptotic [37], and immunomodulatory [38] actions that can be exhibited in the chimeric molecule. Thus, it can be expected that the creation of an IFN-ApoA-I chimera will combine the antiviral activity of IFN with the pharmacokinetic and immunomodulatory benefits of ApoA-I.

In this work, the genes encoding mature human IFN and ApoA-I were designed and synthesized, on the basis of which, using genetic engineering methods, recombinant strains of *Pichia pastoris*—producers of two forms of IFN, both wild-type and chimeric— fused with human apolipoprotein A-I were constructed. The chimeric cytokine consisted of a full-length mature IFN located in the N-terminal region of the polypeptide chain and human ApoA-I in the C-terminal region linked via a flexible oligopeptide linker. The yeast *P. pastoris* was chosen as a producer microorganism because it has a number of advantages over other expression systems: simplicity of genetic manipulation, lack of endotoxin production, a high yield of the target protein, and secretion into the culture fluid, as well as the presence of correct post-translational modifications [39]. Recently, these producers have been widely used for the production of various enzymes [40], therapeutic proteins [41], and vaccines [42]. It was expected that the resulting chimera would have the biological activity characteristic of authentic IFN but a longer action and less cytotoxicity, both due to the presence of ApoA-I in its composition and due to correct glycosylation.

## 2. Results

### 2.1. Creation of Recombinant Plasmids Encoding an Authentic and Chimeric Form of Mature Human IFN

**Figure 1:**
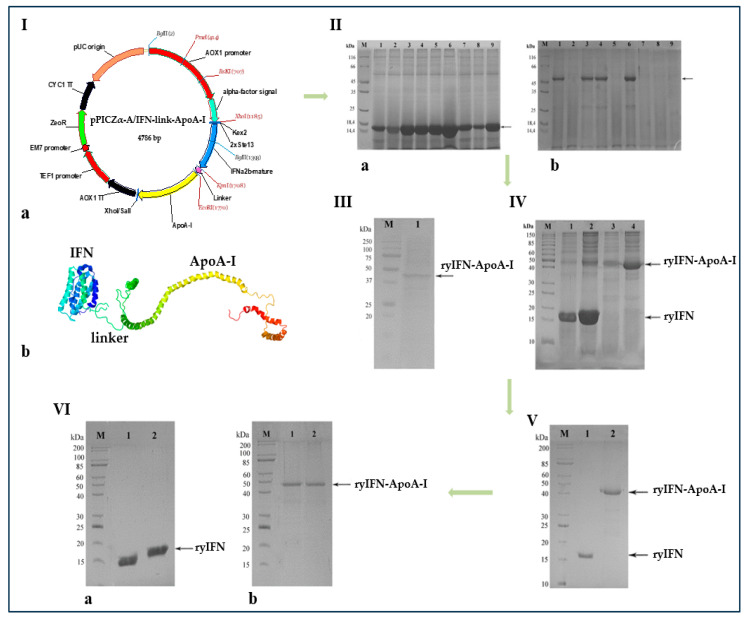
Biosynthesis of the genetically engineered protein IFN-ApoA-I. **I.** Genetic map of pPICZα-A/IFN-link-ApoA-I recombinant plasmid (**a**) and representation a hypothetical tertiary structure of the chimeric protein ryIFN-ApoA-I (**b**). **II.** SDS-PAGE analysis of TCA precipitated proteins from culture supernatants of *P. pastoris* clones expressing ryIFN (**a**) and ryIFN-ApoA-I (**b**) when cultured in a 96-deep-square-well plate. Lane M—standard protein molecular weight marker (14–116 kDa) (Thermo Fisher Scientific); lanes 1–9—TCA precipitated proteins from culture supernatants of nine clones after 96 h induction with 1.0% (*v*/*v*) methanol. **III.** Western blot analysis of proteins from the supernatant of the *P. pastoris* culture using clone #6. Lane M—the pre-stained standard molecular weight marker (15–250 kDa) (Bio-Rad). Lane 1—ryIFN-ApoA-I expressed by *P. pastoris*. **IV.** SDS-PAGE analysis of TCA-precipitated proteins from supernatants of yeast cultures after 4 days of fermentation in conical baffled flasks in the presence or absence of 0.2% *v*/*v* Tween 20. Proteins in the samples of supernatant were concentrated 15 times. Lane M—standard molecular weight marker (10–200 kDa); lanes 1,2—proteins from the supernatants of yeast cultures carrying the plasmid pPICZαA/IFN growing in the absence (1) or presence (2) of 0.2% *v*/*v* Tween 20; lanes 3,4—proteins from the supernatants of yeast cultures carrying the plasmid pPICZα-A/IFN-link-ApoA-I growing in the absence (3) or presence (4) of 0.2% *v*/*v* Tween 20. **V.** SDS-PAGE analysis of purified ryIFN (1) and ryIFN-ApoA-I (2). Lane M—standard molecular weight marker (Sib Enzyme) (10–200 kDa); **VI.** SDS-PAGE analysis of purified ryIFN (**a**) and ryIFN-ApoA-I (**b**) under reducing (1) and non-reducing (2) conditions. Lane M—standard molecular weight marker (10–200 kDa) (Sib Enzyme).

All processes for the construction and isolation of the recombinant plasmids pPICZα-A/IFN and pPICZα-A/IFN-link-ApoA-I are described in the Materials and Methods section. This work included the following steps: (a) prediction of the tridimensional structure of the chimeric construct, (b) constructing synthetic genes for mature human ApoA-I and two variants of mature human IFN (IFN and IFN-linker) and optimization of them for efficient expression in the yeast *P. pastoris*, (c) synthesis and cloning of the optimized two variant IFN and ApoA-I genes in the vectors pD912-AKS and pJ201, respectively, and (d) assembly of the recombinant plasmids pPICZα-A/IFN and pPICZα-A/IFN-link-ApoA-I and transformation of *E. coli* cells with them, followed by isolation and purification for the next stage, i.e., cloning in the yeast *P. pastoris* str. X33. The genetic map of the recombinant plasmid pPICZα-A/IFN-link-ApoA-I is shown in Figure 1I(a).

For a fusion protein, it should be confirmed that the three-dimensional structures and bioactivities of the two proteins have not been substantially affected by each other after fusion. Figure 1I(b) shows a prediction of the tridimensional structure of a chimeric protein calculated using the RaptorX protein structure prediction server (http://raptorx.uchicago.edu, server access date 2 February 2019). As can be seen, the constituent components of the chimeric protein are spatially separated from each other by a linker, thus the functions of IFN and ApoA-I can be expected to be retained in the chimera. As suggested by the predicted tridimensional structure, the two components of the chimeric protein should be able to correctly fold.

### 2.2. Transformation of P. pastoris Cells with Recombinant Plasmids and Screening of Transformants

Selected zeocin-resistant transformants were evaluated for their ability to synthesize and secrete ryIFN and ryIFN-ApoA-I recombinant proteins by culturing them in 96-well plates followed by analysis of the protein spectra in culture supernatants using SDS-PAGE. All analyzed clones bearing the pPICZα-A/IFN plasmid secreted proteins with a molecular mass of ~19 kDa, which corresponds to the calculated molecular mass of IFN; however, the level of protein synthesis was significantly different (Figure 1II(a)). A similar analysis of clones bearing the pPICZα-A/IFN-link-ApoA-I plasmid showed that only some of them synthesize proteins close in molecular weight to the calculated molecular weight of the chimeric protein (Figure 1II(b)). T he level of protein synthesis in these clones was also different and, in general, was significantly lower than in the case of authentic ryIFN. Clones producing the largest quantity of proteins with molecular weights close to the calculated molecular weights of the target proteins ryIFN and ryIFN-ApoA-I were used for further studies. The presence of ApoA-I in the ryIFN-ApoA-I chimeric protein was confirmed by Western blotting using rabbit anti-ApoA-I IgG (Figure 1III).

### 2.3. Fermentation and Purification of ryIFN and ryIFN-ApoA-I

Selected *P. pastoris* clones producing the highest amount of ryIFN (clone #6) and ryIFN-ApoA-I (clone #6) were used for semi-quantitative production of these proteins in conical flasks with deflectors in BMGY medium on an orbital shaker at 25 °C and 250 rpm. At the end of the induction, yeast cells were removed by centrifugation and proteins from the supernatants were precipitated via the addition of dry ammonium sulfate. Before adding sulfate, aliquots of culture broths containing ryIFN and ryIFN-ApoA-I were taken to assess the quantitative yield of these proteins. Aliquots of 300 μL were taken; the proteins from the aliquots were precipitated with TCA and dissolved in 20 μL of sample buffer before being analyzed using SDS-PAGE.

The approximate protein yield was estimated via a densitometric method using the Gel-Pro analyzer 3.0 software. According to the densitometry data, Tween 20 significantly increased the production of the recombinant proteins (Figure 1IV). In the case of ryIFN it was about 120 mg/L and in the case of ryIFN-ApoA-I it was 30 mg/L.

Protein precipitates obtained after precipitation with ammonium sulfate were used for subsequent chromatographic purification of the recombinant cytokines ryIFN and ryIFN-ApoA-I. The precipitated ryIFN was purified via two-step chromatography on SP and DEAE-Sepharose resins. The final recovery of ryIFN was 67% of the amount of protein obtained from the shake flask. The purity of the final preparation was 95–98%. The ryIFN-ApoA-I chimeric protein was purified via reverse-phase chromatography and the final purity was approximately 90–93% (Figure 1V). The difficulties associated with the purification of the chimeric protein can be explained by its nature, in particular by the presence in its composition of the amphiphilic protein ApoA-I, which contains both hydrophobic and hydrophilic regions. As a result, the final recovery of the chimera was 48% of the amount of protein obtained from the shake flask.

Heterologous expression of authentic and chimeric IFN genes can be accompanied by the formation of dimeric and multimeric molecules due to the formation of intermolecular disulfide bonds. To determine the possible presence of covalently cross-linked molecules in ryIFN and ryIFN-ApoA-I preparations, both preparations were analyzed using SDS-PAGE under reducing and non-reducing conditions. As shown in Figure 1VI(a,b), both IFN preparations are clearly composed of appropriate single bands. Therefore, both ryIFN and ryIFN-ApoA-I consist mainly of monomeric forms.

### 2.4. Antiviral Activity of ryIFN and ryIFN-ApoA-I

The specific antiviral activity was determined according to the standard IFN protocol. Like the IFN standard, recombinant ryIFN and its chimeric form with ApoA-I had increased antiviral activity in a dose-dependent manner. It was found that the activity of ryIFN was 1.5 × 10^8^ IU/mg and the activity of ryIFN-ApoA-I–1.6 × 10^8^ IU/mg, values that correspond to the *European Pharmacopoeia* standard. This determination of antiviral activity was carried out in collaboration with the accredited laboratory “Vector Medica” (Novosibirsk, Russia) using pharmacopoeia standards and is based on the determination of cell monolayer damage.

**Figure 2:**
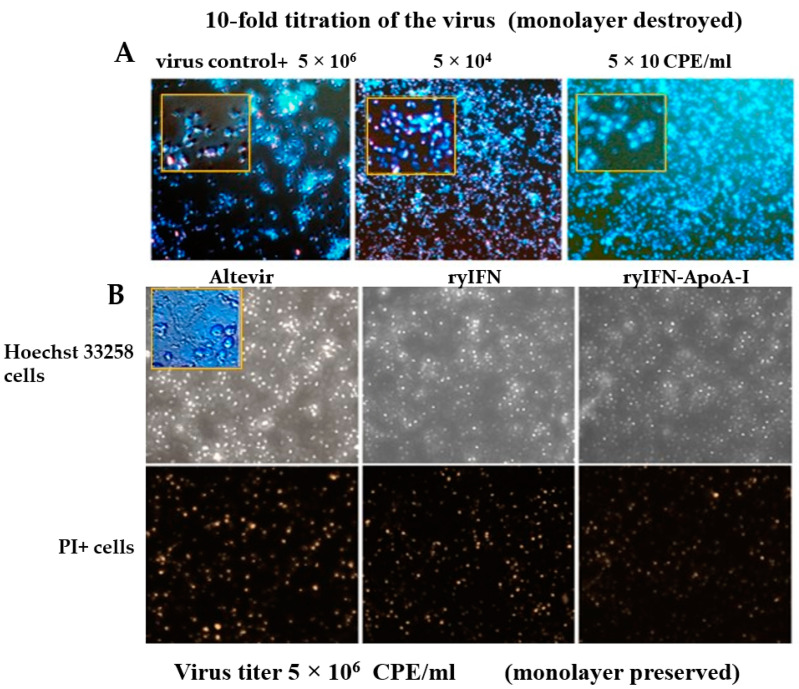
Apoptotic and necrotic patterns of the death of Vero cells infected with SARS-CoV-2. Zoomed-in image of each panel. (**A**) Control samples and virus titers of 5 × 10^6^, 5 × 10^4^, and 5 × 10 CPE/mL. (**B**) The fluorescence signals of Hoechst 33258 and PI in the infected Vero cells were counted 5 days post-infection using 5 × 10^6^ CPE/mL SARS-CoV-2. Zoomed-in image of “Altevir”-preserved monolayer.

To analyze the antiviral activity of the obtained cytokines, we also used the Vero cell culture; Vero cells have an increased number of receptors for the SARS-CoV-2 virus [43] and do not have the proper level of interferon expression [44]. We used fluorescent Hoechst 33258 and propidium iodide (PI) staining of cells to quantify both the apoptotic cell death pathway and the necrotic pathway to look for differences between cytokines (Figure 2).

Infection of the Vero cell culture monolayer with 5 × 10^6^ CPE/mL SARS-CoV-2 resulted in the full destruction of the monolayer with cell detachment in the control sample. Infection with 5 × 10^4^ CPE/mL SARS-CoV-2 led to the destruction of the monolayer with partial detachment of cells and massive cell death, mainly via permeabilization of the cell membranes. A decrease in the virus titer to 50 CPE/mL also led to the disruption of the monolayer and apoptotic cell death that left a small number of cells alive (15.6 ± 7.78%) (Figure 2A).

Type 1 interferons block the synthesis of viral proteins and suppress some other stages of their production, exerting a pronounced antiviral effect. Infection of the Vero cell monolayer with 5 × 10^6^ CPE/mL SARS-CoV-2 in the presence of 100 ng/mL of one of the tested cytokines (“Altevir”, ryIFN, or ryIFN-ApoA-I) in the culture medium contributed to the preservation of the cell monolayer (Figure 2B). After 5 days of cell infection, the monolayers stained with the fluorescence dyes used were observed and counted in the visual fields for PI+ cells and Hoechst 33258+++ cells (Figure 2B). Thus, the leading type of cell death when using ryIFN-ApoA-I was the apoptotic cell death pathway, which accounted for 29.6 ± 3.8% of all cells versus 15.5 ± 3.9% for necrotic cells, with a predominance of living cells 54.9 ± 7.6%. When the culture medium contained ryIFN, the number of apoptotic cells was somewhat higher (33.6 ± 3.9%) and the number of necrotic cells was also (19.6 ± 2.7%). “Altevir” protected cells from death somewhat worse and a high level of the fluorescence of Hoechst 33258+++ was observable in 35.8 ± 4.1% of cases, and PI was observable in 23.8 ± 2.7% of cases, which is statistically significantly different from ryIFN-ApoA-I (*p* < 0.01). In the case of the ryIFN-ApoA-I, the total cell counts per field of vision were higher (345 ± 26) than with the ryIFN (301 ± 17) and “Altevir” (256 ± 19) treatments (Mann–Whitney test, *p* < 0.05). Thus, the studied recombinant cytokines exhibit pronounced antiviral activity.

### 2.5. Cytotoxic Effect of Recombinant Interferons

**Figure 3:**
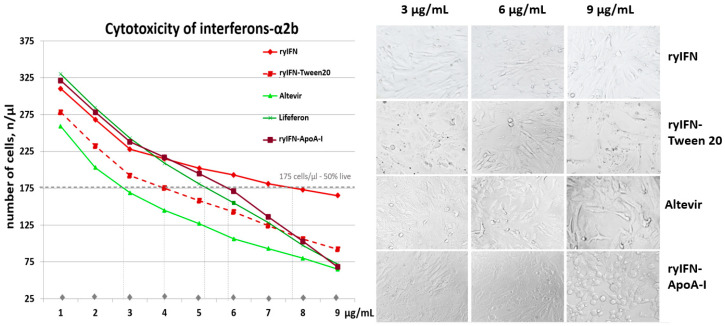
Cytotoxic effect of recombinant IFN-α2b on Vero cells. On the left is the determination of the IC50s for the obtained recombinant interferons (ryIFN and ryIFN-ApoA-I) and commercial preparations of IFN-α2b (“Altevir” and “Lifeferon”). On the right are representative images obtained using a live imaging system demonstrating cell monolayer disruption in the presence of interferon preparations containing non-ionic detergents (“Altevir” containing Tween 80 and ryIFN-ApoA-I containing Tween 20) and ryIFN without Tween 20 (40×).

An analysis of the cytotoxicity of the obtained recombinant interferons and commercial preparations of this cytokine showed that even at a concentration of 1 μg/mL, their toxicity manifests itself. In [45], the 50% inhibitory concentration (IC50) for IFN was 1 µg/mL in the Vero cell line. However, in our experiment, the 50% inhibitory concentration (IC50) for ryIFN was 7.7 ± 0.75 µg/mL, whereas for the commercial standard “Lifeferon” this figure was 5.2 ± 0.5 µg/mL (Mann–Whitney test, *p* < 0.05). The inhibitory concentrations (IC50s) of Altevir containing Tween 80 and ryIFN-ApoA-I containing Tween 20 were 2.8 ± 0.4 μg/mL and 5.8 ± 0.45 μg/mL, respectively. It was found that ryIFN exhibited different cytotoxicity when added to cells in buffers containing Tween and those without it. The IC50 for ryIFN containing 0.03% Tween 20 was 4.0 ± 0.45 µg/mL, whereas for ryIFN without this detergent it was 7.7 ± 0.75 μg/mL (Mann–Whitney test, *p* < 0.05) (Figure 3).

Toxicity was detected at a concentration of 0.03% Tween 20, and in the presence of 0.06% Tween 20, the rounding of cells and detachment from the matrix occurs. Tween 80 also showed toxicity to Vero cells, reducing the total number of cells and causing their apoptotic death. The cells decreased in size and had a pronounced striation, which is explained by the condensation of intracellular organelles during apoptosis (Figure S1). Thus, a significant contribution to the toxic effect on cells was made by the non-ionic detergents that are often included as a stabilizer in protein pharmaceutical compositions. The reference preparation “Altevir” contained Tween 80, and the morphological pattern of cell death at an “Altevir” concentration of 9 μg/mL was similar to the picture when 0.06% Tween 80 was added to the culture medium. The ryIFN-ApoA-I preparation contained Tween 20, which was introduced at the stage of chimeric protein production and during its chromatographic purification. RyIFN-ApoA-I at a concentration of 9 μg/mL sharply reduced cell viability, and the cell death pattern clearly resembled the cell death pattern when using 0.06% of the non-ionic detergent Tween 20 (Figure 3 and Figure S1). Based on the experiments performed, we assume that we cannot completely eliminate the presence of Tween in the ryIFN-ApoA-I preparation at the purification stages and control its exact concentration since the amphipathic nature of the ApoA-I protein may facilitate the binding of Tween 20.

Thus, the IC50 of the commercial drug “Lifeferon” obtained via biosynthesis in *E. coli* is achieved at a lower concentration compared with ryIFN synthesized in *Pichia pastoris*. Both of these preparations did not contain non-ionic detergents in their composition. As can be seen in Figure 3, ryIFN-ApoA-I containing Tween 20 has a titration curve similar to “Lifeferon” and the IC50 is slightly higher for the chimeric protein.

### 2.6. Pharmacokinetics of ryIFN and ryIFN-ApoA-I

**Figure 4:**
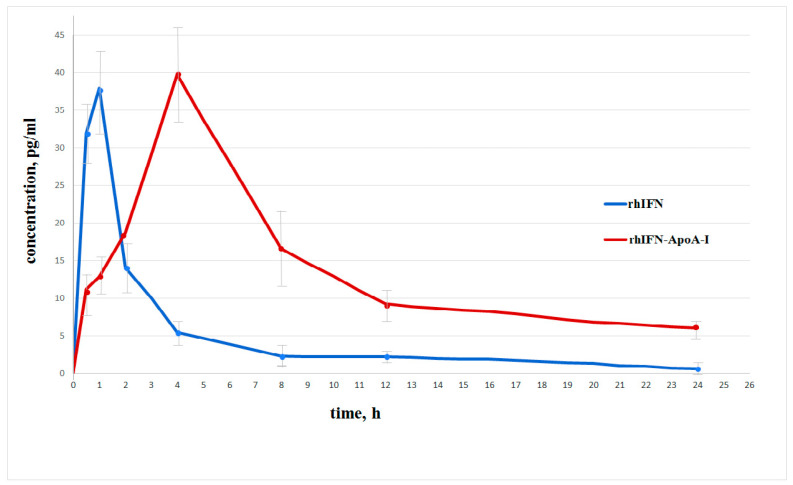
Pharmacokinetic curves of the ryIFN and ryIFN-ApoA-I concentrations in blood plasma after a single subcutaneous injection of them into male CD-1 mice at doses equivalent to their equal molar doses, i.e., 10 μg/kg and 25 μg/kg, respectively. Numerical data are presented as mean ± standard deviation (*n* = 5).

We compared the pharmacokinetic parameters of ryIFN and its chimeric form after a single subcutaneous injection in male CD-1 mice. The results of the pharmacokinetic analysis of the recombinant chimeric cytokine ryIFN-ApoA-I are shown in Table 1. Pharmacokinetic curves of the concentrations of the recombinant proteins after a single subcutaneous injection into male CD-1 mice are shown in Figure 4.

As shown in Table 1, after a single subcutaneous dose of 10 μg/kg ryIFN, the plasma ryIFN concentration peaked 1 h after administration (Cmax = 37.897 ± 4.715 pg/mL), whereas when using 25 μg/kg ryIFN-ApoA-I the maximum concentration is observed after 4 h (Cmax = 39.771 ± 6.645 pg/mL).

In addition, the plasma levels of ryIFN had decreased significantly 8 h after injection (3 pg/mL quantification limit) and, when the chimeric protein was administered, even after 24 h rather high values of 6.045 μg/kg were determined. The obtained AUC0-∞ for ryIFN was 118.140 pg×h/mL and for ryIFN-ApoA-I it was 445.881 pg×h/mL. The AUC0-∞ is an important parameter that determines the degree of absorption of the dosage form.

The calculated value of MRT0-inf, which corresponds to the average retention time of the active substance in the body, was 6.011 h for ryIFN and 16.296 h for ryIFN-ApoA-I. This means the duration that the protein can exhibit its therapeutic properties is 2.7 times longer for the chimeric protein. These results highlight the potential of this chimera to prolong the pharmacological effects of interferon.

## 3. Discussion

IFN is used clinically for the treatment of a wide range of diseases; however, its high toxicity and short half-life in the body significantly limit its use. In our study, we used the strategy of IFN fusion with the blood plasma protein apolipoprotein A-I to increase the half-life of this cytokine and reduce the cytotoxicity of the drug. Chimeric genes encoding full-length human IFN and ApoA-I were optimized for expression in *P. pastoris* cells. One of the main advantages of the methylotrophic yeast *P. pastoris* over bacterial expression systems is the inherent ability to secrete synthesized heterologous proteins into the culture fluid and glycosylate them in much the same way as in humans. The yields of ryIFN and ryIFN-ApoA-I were about 120 mg/L and about 30 mg/L, respectively, in an uncontrolled flask culture. This study is not the first time that we have been faced with a low level of synthesis of ApoA-I and chimeric proteins containing ApoA-I [46]. Although genes for chimeric proteins were engineered and optimized for expression in yeast, the yield of the secreted chimeric proteins varied significantly [47,48]. The yield of proteins could most likely be significantly increased by carrying out cultivation in a bioreactor. We will use this method of growing yeast in subsequent works. It is also possible that, during the cultivation of the producer strain, both ApoA-I [46] and ApoA-I-containing proteins cause partial lysis of producer cells due to the membranotropic action of ApoA-I, as a result of which the total yield of the target protein will decrease.

It is noteworthy that the addition of Tween 20 to the culture medium in the induction phase allowed a significant increase in the yield of both ryIFN and its chimera (by approximately a factor of three). The addition of Tween 20 has had a positive effect on the production yields of other recombinant yeast proteins obtained by us (G-CSF and G-CSF-ApoA-I [48,49]; GM-CSF and GM-CSF-ApoA-I [47]). There are a lot of data on the effect of non-ionic detergents not only increasing the secretion of target proteins but also increasing the stability of the protein in solution and preventing its aggregation [50,51]. Thus, non-ionic detergents are often present in pharmaceutical preparations [52].

The biological activity (antiviral activity) of yeast-derived cytokines was evaluated against both the vesicular stomatitis virus and the SARS-CoV-2 virus. These models demonstrated complete retention of the antiviral activity of the chimeric form of IFN. The specific biological activity of the chimera did not decrease compared with authentic ryIFN, although, according to the literature, chimeric cytokines often have significantly lower activity due to a stereospecific blockade of the molecule that leads to difficulty in binding to receptors [16,53]. Analysis of the integrity of the Vero cell monolayer upon infection with SARS-CoV-2 showed the effective antiviral activity of the recombinant cytokines we obtained. However, the use of fluorescent dyes to determine the pathway of cell death (apoptosis or necrosis) also showed that in the presence of ryIFN-ApoA-I there was an increased number of living cells and a decrease in the number of necrotic cells compared with a standard drug. ApoA-1 appears to contribute by positively affecting cell survival in this design. We previously observed a similar effect in experiments with other chimera [47]. The ability of apoA-1 to increase cell survival has been demonstrated elsewhere [54]. It should be noted that, in the presence of ryIFN-ApoA-I, single necrotic foci were sometimes observed in the area of an increased number of cells.

To assess the cytotoxicity of recombinant proteins, commercial α2-b interferons were taken as reference standards. “Altevir” contained a non-ionic detergent in its composition, whereas “Lifeferon” did not. The stabilizing effects of surfactants are associated with preferential adsorption at the interface and inhibition of protein–protein interactions that lead to a decrease in protein unfolding and the formation of protein particles [55]. At the same time, the cytotoxic effect of surfactants is known [56,57]. Surfactants increase membrane permeabilization, causing membrane disruption and initiating apoptosis. Hua T. et al. [58] found that low concentrations of Tween 20 increase membrane permeabilization and enhance the transduction of PK-15 cells by the PCV2 virus. We also discovered the toxic effects of the non-ionic detergents Tween 20 and Tween 80, which are most often used in various pharmaceutical compositions. Tween 20 at a concentration of 0.06% caused cell death and cell detachment from the plastic. The addition of increasing concentrations of up to 9 μg/mL of ryIFN-ApoA-I to the culture medium was accompanied by an increase in the Tween 20 content to 0.06%, which caused a sharp increase in the cytotoxicity of the drug. However, at a concentration of 6 μg/mL, the IC50 for the chimeric form was higher than that of the reference IFN preparations. With the addition of Tween 20 (0.03%), the nonspecific cytotoxicity of ryIFN increased sharply; the IC50 was reduced by almost a factor of 2 compared with ryIFN, which further emphasized the importance of monitoring the concentration of non-ionic detergents in therapeutic products. However, ryIFN and its chimeric form had a significantly lower cytotoxic effect on cells than the reference commercial drugs “Altevir” and “Lifeferon” in the IC50 region, which was higher for ryIFN and its chimeric form. The lower cytotoxicity can be explained by the antiapoptotic effect of ApoA-I in the chimera containing Tween 20 at 0.03% and the absence of Tween in the ryIFN preparation. It is also possible that the proteins obtained from *P. pastoris* cells are less toxic than those from bacterial expression systems (for example, “Lifeferon”).

ApoA-I in a chimera can modulate the action of the cytokine fused with it, imparting new properties to the chimeric protein, as we showed in earlier work [48]. Previously, Pedro Berraondo et al. created a number of other plasmid constructs encoding ApoA-I fused with the cytokines IL-15 [59], TGF-b [60], and FGF15/19 [61] that were injected into mice. Chimeric proteins synthesized in mice have shown cytokine-specific biological activities with improved pharmacokinetic properties. A plasmid containing IFN-ApoA-I has been shown to have increased immunostimulating properties, reduced hematotoxicity, and better pharmacokinetic characteristics [62].

Thus, it can be expected that the ryIFN-ApoA-I chimera may have additional (or altered) functions compared with authentic ryIFN. In particular, our latest (not yet published) work demonstrated a significantly higher fungistatic effect for ryIFN-ApoA-I compared with the authentic cytokine. The cytotoxicity of interferons is most severe when used frequently, for example in cancer, leading to cognitive impairment, depression, and amyloidosis. This influence of interferons is associated with the activation of interferon-responsive genes, the initiation of inflammation, and the subsequent destruction of the neuronal network [63]. It is worth noting that ApoA-I is involved in the body’s anti-inflammatory strategy and maintains cell viability [64]. It is possible that a chimeric form of interferon containing ApoA-I may exhibit less cytotoxicity towards microglia and the neuronal network. Clarification of this issue is of undoubted interest, but, unfortunately, this work is beyond the scope of this study.

In the present study, we also determined the pharmacokinetic parameters of the ryIFN-ApoA-I chimeric protein in a mouse model. The half-life of the chimera was found to be 1.8 times greater than that of authentic ryIFN. The average retention time of the chimera in the blood was 2.7 times higher than that of the ryIFN and the time to reach the maximum concentration in the blood was 4 h; for the ryIFN this value was 1 h. Thus, it can be concluded that the chimeric ryIFN enters the bloodstream more slowly and has a longer half-life in the body. Perhaps its effect will be milder if longer use is required.

Thus, the results presented in this study, along with the published data from other researchers, indicate that ApoA-I-containing chimeric forms of cytokines are of undoubted interest. They can be positioned not only as molecules with a prolonged action of cytokines but also as possible new therapeutic agents that can be used against the background of various disorders of the body’s functions.

## 4. Materials and Methods

### 4.1. Materials

All chemical reagents were of analytical grade and purchased from either Sigma-Aldrich (USA) or Reachim (RF). The international standard interferon alpha 2b, (MCO1) code: 95/566 was the reference drug for assessing the antiviral activity of recombinant interferons. Restriction endonucleases were purchased from SibEnzyme (Novosibirsk, Russia). T4 DNA ligase and Phusion DNA polymerase were purchased from Thermo Fisher Scientific Inc., (Carlsbad, CA, USA). Oligonucleotides were purchased from LTD “Biosynthesis” (Novosibirsk, Russia). Yeast extract, bacto pepton, and tripton were purchased from Difco (BD Life Sciences, Franklin Lakes, NJ, USA). and were used for preparations of Luria-Bertani (LB) medium for the growing of *E. coli*. Igla media MEM Biolot (St. Petersburg, Russia), fetal bovine serum HyClone (South Logan, UT, USA), benzylpenicillin, and streptomycin were purchased from Gibco Thermo Fisher Scientific Inc., (USA), TrypLE Gibco Thermo Fisher Scientific, (UK). The fluorescent dyes Hoechst 33258 and propidium iodide (PI) Sigma-Aldrich, (St. Louis, MO, USA) were used. DEAE-Sepharose FF and SP Sepharose FF ion-exchange resins were purchased from GE Healthcare Bioscience (Uppsala, Sweden), SupelpakTM-2SV (Sigma-Aldrich, Supelco, Bellefonte, PA, USA). Nitrocellulose membranes were purchased from Merck, Millipore (Luxembourg, USA). The water used in the work was deionized and autoclaved.

### 4.2. Bacterial and Yeast Strains and Plasmid Vectors

*Escherichia coli* str. TOP10, *Pichia pastoris* str. X33, and the pPICZα-A vector were purchased from Invitrogen Inc. (Waltham, MA, USA).

### 4.3. Construction of the Recombinant Plasmids pPICZα-A/IFN and pPICZα-A/IFN-link-ApoA-I

The nucleotide sequences of synthetic human genes encoding mature ApoA-I, IFN, and IFN genetically fused at its C-terminus to the N-terminus of a GSSGSGGSSGSGSGSSGGSG linker were designed and optimized for expression in *P. pastoris* yeast using the computer program Gene Designer (ATUM, USA) and the VisualGeneDeveloper 1.9 software package (http://www.visualgenedeveloper.net/Download.html accessed on 2 February 2019).

The gene encoding mature human ApoA-I was flanked at the 5′ end by an EcoRI restriction site and at the 3′ end by a translation termination codon and an XhoI restriction site. The gene coding for mature IFN was flanked at the 3′ end by a translation termination codon followed by a SalI restriction site. The 5′ region of the gene contained a XhoI restriction site and a sequence encoding sites for hydrolysis by the proteases Kex2 and Ste13 in the 5′–3′ direction. The second variant of the IFN gene (IFN-linker) was similar to the first; however, the coding region of mature IFN was followed by a KpnI site, a sequence coding for a flexible glycine–serine linker (GSSGSGSSGSGSGSSGGSG), and EcoRI and SalI sites in the 5′–3′ direction. Optimized genes encoding IFN, IFN-linker, and ApoA-I were synthesized by ATUM (USA) and cloned in *E. coli* into the plasmids pD912-AKS or pJ201. Both IFN genes were excised from the plasmids pD912-AKS/IFN and pD912-AKS/IFN-linker at the XhoI and SalI sites and inserted into the pPICZα-A vector at the same sites. Temperature-cycle ligation using T4 DNA ligase was carried out as described in [65].

### 4.4. Transformation of Electrocompetent E. coli Cells and Screening of Transformants

The resulting ligase mixtures were used to transform TOP-10 *E. coli* cells as described previously [49]. Colony PCR was used to select transformants containing the recombinant plasmids. PCR was performed using a BIS DNA thermal cycler (BIS, Russia) in the presence of a pair of primers: forward primer 326-F (5′-TACTATTGCCAGCATTGCTGC-3′) and reverse primer 325-R (5′-GCAAATGGCATTCTGACATCC-3′). The PCR-positive colonies were used for recombinant plasmid isolation.

The gene coding for mature human ApoA-I was excised from the pJ201/ApoA-I plasmid at the EcoRI and XhoI sites, inserted into the pPICZα-A/IFN-linker vector at the same sites, and ligated as described above. The ligase mixture was used to transform TOP-10 *E. coli* cells. A PCR-positive colony containing the plasmid pPICZα-A/IFN-link-ApoA-I was used to isolate the plasmid by alkaline lysis. The isolated plasmids pPICZα-A/IFN and pPICZα-A/IFN-link-ApoA-I were subsequently cloned into *P. pastoris* cells.

### 4.5. Transformation of Electrocompetent P. pastoris str. X33 Cells and Transformant Screening

*P. pastoris* str. X33 electrocompetent cells were prepared according to the manufacturer’s instructions (Invitrogen, USA) and transformed with 5–10 μg of plasmid DNA (pPICZα-A/IFN or pPICZα-A/IFN-link-ApoA-I) before being preliminary linearized by digestion with the restriction endonucleases SacI or BstXI. Transformation and screening of the transformants to produce target proteins were carried out as described previously [47]. The clones producing the maximum amounts of proteins corresponding to the molecular weights of authentic ryIFN or of chimera ryIFN-ApoA-I were used in subsequent work. The presence of ApoA-I in the ryIFN-ApoA-I chimera was confirmed by Western blotting using rabbit anti-ApoA-I IgG.

### 4.6. Cultivation of P. pastoris Clones Producing Recombinant ryIFN and ryIFN-ApoA-I

Selected *P. pastoris* clones that produced proteins corresponding in size to the target recombinant cytokines ryIFN and ryIFN-ApoA-I were inoculated into 10 mL of BMGY medium in a 50 mL conical flask and grown at 28 °C for 48 h at 250 rpm. On the third day, the grown clone cultures were inoculated into 100 mL of BMGY medium in 500 mL baffled conical flasks and the culture was continued at 25 °C and 250 rpm for the next 48 h. On the fifth day, Tween 20 and methanol were added to the flasks to final concentrations of 0.2% (*w*/*v*) and 1% (*v*/*v*), respectively, and then only methanol was added daily for 4 days up to a final concentration 1%.

Upon completion of the cultivation, the cell cultures were centrifuged at 3750× *g* and 4 °C for 30 min. The cell pellet was discarded, and ammonium sulfate powder was added to the selected supernatants to 40% saturation in the case of ryIFN and to 50% saturation in the case of ryIFN-ApoA-I at +4 °C. The suspensions were incubated overnight at +4 °C. Protein precipitates were precipitated by centrifugation at 39,000× *g* for 30 min at +4 °C.

### 4.7. Isolation and Purification of Recombinant Proteins

(a) RyIFN purification

Protein precipitates containing mainly ryIFN, after precipitation with ammonium sulfate, were dissolved in buffer “A” containing 25 mM sodium acetate and 1 mM EDTA, pH 4.5. The undissolved part of the protein was precipitated by centrifugation at 37,000× *g* for 20 min at +4 °C. The clarified supernatant was loaded onto a DEAE Sepharose FF column equilibrated with buffer “A”. The column was washed with buffer “A” and the part unbound to the resin material was collected and loaded onto an SP-Sepharose FF column equilibrated with buffer “A”. The column was washed with the same buffer and the protein was eluted using a linear gradient of sodium chloride (0–0.5 M) in buffer “A”. The maximally purified fractions were pooled and dialyzed against buffer B (10 mM sodium phosphate, pH 7.5, 1 mM EDTA). The dialyzed solution was loaded onto a DEAE Sepharose FF column equilibrated with buffer “B”. The column was washed with the same buffer and the protein was eluted using a linear gradient of sodium chloride (0–0.25 M) in buffer “B”. The fractions were analyzed by SDS-PAGE and those containing the most purified ryIFN were pooled.

(b) RyIFN-ApoA-I purification

The chimeric protein was purified using reverse-phase chromatography on SupelpakTM-2SV resin (Supelco). Protein precipitates formed after the addition of ammonium sulfate were dissolved in 0.03% Tween 20. The undissolved part of the protein was precipitated by centrifugation at 37,000× *g* for 20 min at +4 °C. Trifluoroacetic acid (TFA) was added to the clarified supernatant to a final concentration of 0.2% and then the supernatant was loaded onto a chromatographic column with 10 mL of SupelpakTM-2SV resin. The column was washed with a solution containing 20% acetonitrile and 0.1% TFA. The target protein was eluted from the column with a solution containing 0.1% TFA and increasing amounts (gradient) of acetonitrile (20–80%). All fractions were analyzed by electrophoresis in 12% SDS-PAGE. Fractions containing the most purified chimeric protein were pooled and dialyzed against buffer “B”.

### 4.8. Analysis of ryIFN and ryIFN-ApoA-I by SDS-PAGE under Reducing and Non-Reducing Conditions

The analysis of purified ryIFN and ryIFN-ApoA-I preparations for the possible presence of covalently “cross-linked” forms was performed via the electrophoresis of proteins under reducing and non-reducing conditions. Protein samples were boiled for 5 min in 4× SDS gel loading buffer containing and not containing 5% (*v*/*v*) β-MeEtOH and subjected to electrophoresis on 12% SDS-PAGE. Proteins in the gel were stained with Coomassie blue.

### 4.9. Determination of the Antiviral Activity of ryIFN and ryIFN-ApoA-I

(a) Antiviral activity against the equine vesicular stomatitis virus

The antiviral activities of IFN and its chimeric form were determined by their abilities to inhibit the cytopathic effect caused by the equine vesicular stomatitis virus (VSV, GKV No. 600, Indiana strain deposited in the State Virus Collection of the D.I. Ivanovsky) at a dose corresponding to 100 TCID50/0.1 mL on a transplantable bovine kidney cell line (MDBK). The reference drug was the international standard sample of human alpha-2 interferon (MCO1) code: 95/566. MDBK cells in Eagle’s MEM were counted and 2 × 105 cells/mL were seeded into a 96-well plate and incubated for 24 h at 37 °C and 5% CO_2_. A series of dilutions of the experimental samples of IFN and a reference preparation (MSO) were added to the plate, then the adherent cells were infected with a virus of known titer. The plate was incubated under the same conditions for 24 h. The plaques formed by the accumulation of the virus were counted under a microscope and expressed in units of plaque formation per mL. One activity unit was defined as the amount of IFN required to obtain the equivalent antiviral activity and is expressed in standard international units. Each sample was tested in triplicate. The virus infectious titer was calculated using the Spearman–Karber formula.

(b) Antiviral activity against SARS-CoV-2

The SARS-CoV-2 initial variant used in this work was obtained by the authors [66]. The continuous culture of Vero cells was cultivated in 48-well plates in Eagle’s MEM supplemented with 10% fetal bovine serum to a monolayer culture. Then, the test cytokine (ryIFN, ryIFN-ApoA-I, or a reference drug (Altevir, Pharmstandard, RF)) was added to the wells of the plate at a concentration of 100 ng/mL. At the same time, monolayers with introduced cytokines were infected with the SARS-CoV-2 virus with specified titers and cultivated for 5 days. The initial virus titer was 5 × 10^6^ CPE/mL and 10-fold dilutions up to 5 × 10 were used. On day 5 after infection, the medium was supplemented with the dye Hoechst 33258 at a final concentration of 5 µg/mL and exposed for 20 min to detect the nuclei of apoptotic cells. To visualize necrotic cell death, propidium iodide was added 15 min after the addition of the Hoechst 33258 dye. Each sample was tested in triplicate. Fluorescence (Hoechst and PI) was recorded for each well with an Axio Observer Z1 (Zeiss, Jena, Germany) at ×10, ×20, and ×40 magnifications. The percentage of nuclei with condensed chromatin (apoptotic cells) and stained with PI (necrotic cells) was calculated relative to the total number of cells.

### 4.10. Determination of the Cytotoxicity of ryIFN and ryIFN-ApoA-I

Vero cells were cultivated in 48-well plates to a monolayer culture. The test cytokine (ryIFN, ryIFN-ApoA-I, or reference drug “Altevir” as a positive control) was added to the plate at concentrations of 1 µg/mL, 3 µg/mL, 6 µg/mL, 9 µg/mL, and 27 µg/mL and Vero cells were cultured for 24 h. Phase-contrast analysis of each well was performed using an Axio Z1 observer. Cells were harvested with TrypLE, resuspended in 100 µL phosphate-buffered saline, and the total number of cells in each well was counted using a CytoFlex S100 flow cytometer (Beckman Coulter, Brea, CA, USA). The analysis was carried out in triplicate. “Altevir” contains Tween 80 and ryIFN-ApoA-I contains Tween 20 at a concentration of 0.03; however, ryIFN and “Lifeferon” do not contain detergent additives. To standardize the experiment, Tween 20 was added to the original ryIFN preparation to a final concentration of 0.03%. First, the cytotoxicity of each detergent (Tween 20 and Tween 80) was evaluated on the Vero cell line in the absence of the test cytokines in the medium.

### 4.11. Pharmacokinetics

The pharmacokinetics of the recombinant chimeric interferon ryIFN-ApoA-I was investigated in comparison with the pharmacokinetics of the authentic human ryIFN. Outbred male CD-1 mice (25–35 g) from the vivarium of the Federal Research Center of the Institute of Cytology and Genetics SB RAS were used for pharmacokinetics studies. The animals were kept under standard vivarium conditions with free access to water and standard pelleted food. Seventy mice were randomized by weight and divided into 2 groups after quarantine. The work with animals was carried out in strict accordance with the Order of the Ministry of Health of the Russian Federation No. 199n dated 1 April 2016 “Good Laboratory Practice” and the provisions of Directive 2010/63/EU of the European Parliament (Directive 2010/63/EC on the protection of animals used for scientific purposes) and of the Council of the European Union of 22 September 2010 on the protection of animals used for scientific purposes.

Cytokines were administered as a single subcutaneous injection at doses of 10 μg/kg ryIFN and 25 μg/kg ryIFN-ApoA-I in phosphate-buffered saline (PBS). Control mice were injected with an equivalent amount of PBS. Blood samples were collected in heparinized tubes at 0.5, 1, 2, 4, 8, 12, and 24 h after protein injection. Five mice were taken at each time point. To obtain plasma, blood samples were centrifuged for 15 min at 3000 rpm. The plasma samples were frozen at −70 °C until analysis. The serum concentrations of ryIFN and ryIFN-ApoA-I were determined using a human interferon alpha ELISA kit Vector Best (Novosibirsk, Russia). Pharmacokinetic analysis was carried out on a tubeless model using the PKSolver program—an add-in for Microsoft Office Excel [67]. The following pharmacokinetic parameters were determined: maximum plasma concentration (Cmax), time to reach Cmax (Tmax), area under the curve between 0 and ∞ (AUC0-∞), and apparent half-life (T1/2).

### 4.12. Statistical Analysis

The maximum concentration (Cmax) and the time to reach Cmax (Tmax) were determined directly from the plasma concentration values. Other pharmacokinetic parameters were calculated using PKSolver, an add-in for Microsoft Office Excel 2007. Statistical data processing was performed using Statistica 6.0 software. Data are presented as arithmetic means and standard error of the mean. Comparisons were performed using the Mann–Whitney U Test. *p* ≤ 0.05 was taken as the level of significance. The data are shown as a mean arithmetic value and standard error of the mean to determine antiviral activity and cytotoxicity.

## 5. Conclusions

This study used protein (apolipoprotein A-I) fusion technology to prolong the half-life of interferon in the blood and reduce cytotoxicity. The chimeric protein was obtained via expression in the methylotrophic yeast *Pichia pastoris*. The chimera fully retained its antiviral activity, contributed to the preservation of cell viability under viral load conditions, and demonstrated an extended half-life compared with authentic IFN. ApoA-I can be positioned as a promising platform for the production of long-acting cytokines with synergistic protein properties.

## 6. Patents

A patent for invention from the Russian Federation, No. 2764787, dated 21 January 2022 “Recombinant plasmid DNA encoding chimeric interferon alpha2b, recombinant yeast strain P. pastoris X33–producer of chimeric interferon alpha2b and a method for producing the specified protein”, authors Beklemishev A.B., Pykhtina M.B, was obtained.

## Figures and Tables

**Table 1:** The pharmacokinetic parameters of ryIFN and ryIFN-ApoA-I after a single subcutaneous injection into male CD-1 mice of their equal molar doses, i.e., 10 μg/kg and 25 μg/kg, respectively.

Protein	Cmax (pg/mL)	SE of Cmax	AUC0-inf_obs (pg/mL × h)	MRT (H)	T1/2 (h)	Tmax (h)
ryIFN	37.897	6.0	4.715	118.140	6.701	1.000
ryIFN-ApoA-I	39.771	16.296	6.645	445.881	12.205	4.000

## Data Availability

All data are available within the manuscript and upon request to the corresponding authors.

## Supplementary Materials

The following supporting information can be downloaded at: https://www.mdpi.com/article/10.3390/molecules28248014/s1, Figure S1: Cytotoxic effect of Tween solvents on Vero cells (40×).
