# Supplementary figures and images for: Engineering a New IFN-ApoA-I Fusion Protein with Low Toxicity and Prolonged Action
Source: Molecules. 2023 Dec 8;28(24):8014. doi: 10.3390/molecules28248014 (PMC10745500; doi:10.3390/molecules28248014)

---

## Supplementary

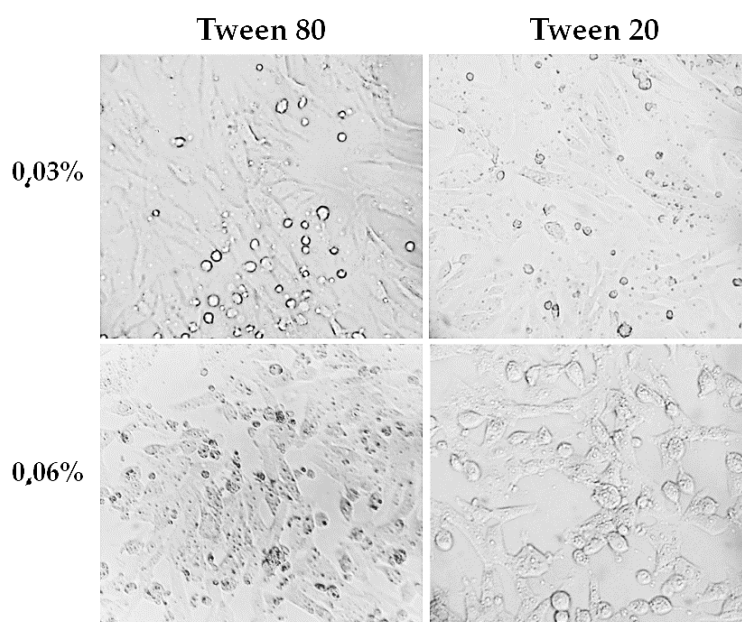

**Figure S1.** Cytotoxic effect of Tweens on Vero cells (40x).

Supplement: Supplementary file 1 [file molecules-28-08014-s001.zip › molecules-2714982-supplementary.pdf]
